# Initial Cell Seeding Density Influences Pancreatic Endocrine Development During *in vitro* Differentiation of Human Embryonic Stem Cells

**DOI:** 10.1371/journal.pone.0082076

**Published:** 2013-12-04

**Authors:** Blair K. Gage, Travis D. Webber, Timothy J. Kieffer

**Affiliations:** 1 Department of Cellular and Physiological Sciences, University of British Columbia, Vancouver, British Columbia, Canada; 2 Department of Surgery, University of British Columbia, Vancouver, British Columbia, Canada; University of Houston, United States of America

## Abstract

Human embryonic stem cells (hESCs) have the ability to form cells derived from all three germ layers, and as such have received significant attention as a possible source for insulin-secreting pancreatic beta-cells for diabetes treatment. While considerable advances have been made in generating hESC-derived insulin-producing cells, to date *in vitro*-derived glucose-responsive beta-cells have remained an elusive goal. With the objective of increasing the *in vitro* formation of pancreatic endocrine cells, we examined the effect of varying initial cell seeding density from 1.3 x 10^4^ cells/cm^2^ to 5.3 x 10^4^ cells/cm^2^ followed by a 21-day pancreatic endocrine differentiation protocol. Low density-seeded cells were found to be biased toward the G2/M phases of the cell cycle and failed to efficiently differentiate into SOX17-CXCR4 co-positive definitive endoderm cells leaving increased numbers of OCT4 positive cells in day 4 cultures. Moderate density cultures effectively formed definitive endoderm and progressed to express PDX1 in approximately 20% of the culture. High density cultures contained approximately double the numbers of PDX1 positive pancreatic progenitor cells and also showed increased expression of *MNX1*, *PTF1a*, *NGN3*, *ARX*, and *PAX4* compared to cultures seeded at moderate density. The cultures seeded at high density displayed increased formation of polyhormonal pancreatic endocrine cell populations co-expressing insulin, glucagon and somatostatin. The maturation process giving rise to these endocrine cell populations followed the expected cascade of pancreatic progenitor marker (*PDX1* and *MNX1*) expression, followed by pancreatic endocrine specification marker expression (*BRN4*, *PAX4*, *ARX*, *NEUROD1*, *NKX6.1* and *NKX2.2*) and then pancreatic hormone expression (insulin, glucagon and somatostatin). Taken together these data suggest that initial cell seeding density plays an important role in both germ layer specification and pancreatic progenitor commitment, which precedes pancreatic endocrine cell formation. This work highlights the need to examine standard culture variables such as seeding density when optimizing hESC differentiation protocols.

## Introduction

Human islet transplantation is a potential cure for type 1 diabetes, although limited cadaveric islet availability precludes widespread clinical application [1,2]. By definition, human embryonic stem cells (hESCs) have the potential to form cells derived from all three embryonic germ layers including endoderm-derived pancreatic endocrine cells. In order to effectively utilize hESCs as a therapeutic source for islet transplantation, highly efficient differentiation of pancreatic endocrine cells must be achieved either *in vitro* or *in vivo* following known developmental cues [3,4]. Based primarily on developmental literature from murine and zebrafish model systems, considerable advances have been made in generating pancreatic endocrine cells from hESCs [5,6]. However, the fundamental differences between human and mouse islet architecture and nutrient responsiveness [7-10] suggests that more empirical optimization may be required to successfully adapt hESC differentiation protocols to human applications [11].

 To date a number of landmark studies have explored the ability to produce functional pancreatic endocrine cells from hESCs both *in vitro* [5,12-15] and *in vivo* [6,16-18]. While *in vivo* maturation of *in vitro* derived pancreatic progenitors has been able to produce pancreatic endocrine cells capable of controlling blood glucose in mice, *in vitro* studies have been far less successful at producing functional endocrine cells. Most *in vitro* studies have used empirical testing of different culture conditions in order to determine the ideal stage-specific differentiation conditions required to convert hESCs to either progenitors or hormone-positive cells. Typically culture conditions have been designed to mimic developmental signalling pathways reported to induce progenitor cell development in various model organisms. Using this approach, the approximately three stage framework for forming pancreatic endocrine competent progenitor cells from hESCs has become TGF-beta signalling (Activin A) dependent induction of definitive endoderm [19,20], FGF7 or FGF10 enhanced patterning to endodermal gut tube [5,6], and retinoic acid dependent induction of PDX1 [5,21,22] with concurrent BMP and sonic hedgehog inhibition [5,14,15,21]. A considerable range of signalling molecules has been applied to coax endocrine cell development from endocrine-competent progenitors; these include exendin-4, IGF1, HGF, noggin, bFGF, BMP4, VEGF, WNT and various inhibitors of sonic hedgehog, TGF-beta, and NOTCH signalling pathways [5,14,23]. 

We sought to examine whether cell seeding density, the first step of any hESC differentiation protocol, might also influence the efficiency of hESC differentiation into pancreatic endocrine cells. Recently even the media buffering component HEPES [17] and the common organic solvent DMSO [24] have been shown to have dramatic effects on pancreatic progenitor and endocrine differentiation purity, suggesting that previously unrecognized components of the hESC differentiation protocol may profoundly impact results. In addition, seeding density has previously been shown to be important during other *in vitro* differentiation models including adipocyte differentiation [25]. Here we seeded cells at four different densities, examined cell cycle progression of undifferentiated cells and tracked the formation of definitive endoderm (CXCR4/SOX17 co-positive cells) followed by pancreatic progenitors (PDX1 positive) and ultimately pancreatic endocrine formation (insulin, glucagon, and somatostatin-positive populations). While efficient definitive endoderm induction was observed above moderate densities of 2.6 x 10^4^ cells/cm^2^, PDX1 expression and subsequent hormone positive populations were increased in cultures seeded at 5.3 x 10^4^ cells/cm^2^. These high seeding density cultures followed the expected temporal expression patterns of maturing pancreatic progenitors that specify endocrine cell fates and finally adopt hormone expression.

## Materials and Methods

### Ethics Statement

This work was approved by the Canadian Stem Cell Oversight Committee and the UBC Clinical Research Ethics Board. 

### Culture of hESCs

Undifferentiated CA1S hESCs kindly provided by the lab of Dr. Jamie Piret (University of British Columbia) and previously published by Caron et al. (2013) [26] were cultured at 37°C, 5% CO_2_ on growth factor reduced-Matrigel-coated (1:30 diluted, BD Biosciences, Mississauga, ON, Canada) plates under feeder-free conditions in mTeSR1 media (STEMCELL Technologies, Vancouver, BC, Canada) as previously described [26]. CA1S cells were enzymatically passaged using Accutase (STEMCELL Technologies) every 2 to 3 days at an approximate split ratio of 1:5 to maintain cells between 20 and 80 percent confluence. WA01 (H1) hESCs, obtained from the WiCell Institute, were cultured similarly as CA1S hESCs on Matrigel-coated plates in mTeSR1 media and passaged using Versene EDTA dissociation solution (Lonza, Walkersville, MD, USA) every 4-5 days.

### Pancreatic Differentiation of hESCs

**Figure 1:**
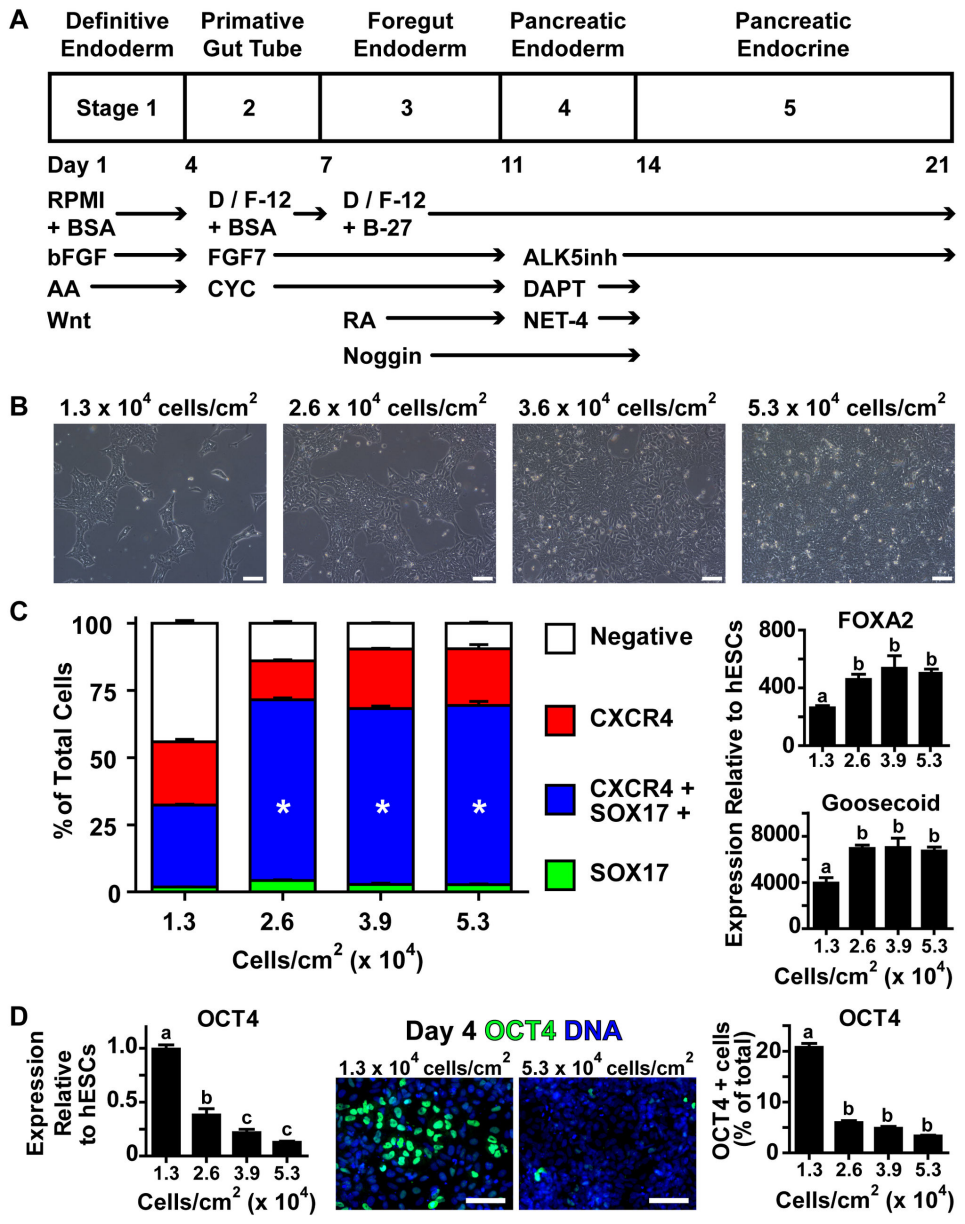
Higher Cell Seeding Density Improves Definitive Endoderm Differentiation. (A) CA1S hESCs were differentiated using a protocol designed to mimic human development in a 21 day, 5 stage process. (B) hESCs were seeded onto matrigel-coated culture plates at the indicated density, yielding 30%-100% confluence as shown at 24 hours after seeding. (C) On day 4 of differentiation, markers of definitive endoderm induction were assessed by flow cytometry (CXCR4 and SOX17 expression) or RT-qPCR (*FOXA2* and Goosecoid, shown relative to undifferentiated hESC expression levels). (D) Expression of OCT4 (marker of pluripotent cells) was assessed by RT-qPCR and immunofluorescence as a percentage of the total number of nuclei (OCT4 is green, nuclei are blue). * represents significant difference from 1.3 x 10^4^ cells/cm^2^ by one-way ANOVA with Bonferroni post-hoc test. Different superscripts (a, b, c) are significantly different from each other within each graph by one-way ANOVA with Bonferroni post-hoc test. Scale bars are 100 μm.

Pluripotent CA1S and WA01 hESCs were seeded onto 12-well culture plates coated with 1:30 diluted growth factor reduced Matrigel at defined densities between 1.3 and 5.3 x 10^4^ cells/cm^2^ for CA1S cells and 2.6 to 10.6 x 10^4^ cells/cm^2^ for WA01 cells in 1.5 ml of mTeSR1 media per well. Cells were enumerated using a Scepter^TM^ 2.0 Automated Cell Counter using 60 μm tips (Millipore, Billerica, MA, USA). Sixteen to twenty hours after seeding for CA1S cells and forty-eight hours after seeding for WA01 cells, differentiation to pancreatic endocrine cells was begun following a previously published 21-day protocol (Figure 1A) known to produce polyhormonal pancreatic endocrine cells in culture with H1 hESCs [18] but until now not tested in CA1S hESCs. Undifferentiated hESCs were exposed to RPMI1640 (RPMI; cat # 61870, Life Technologies, Burlington, ON, Canada) containing 2% fatty acid-free bovine serum albumin (BSA; Proliant, Ankeny, IA, USA), 100 ng/ml activin-A (AA; R&D Systems, Minneapolis, MN, USA), 20 ng/ml Wnt3A (R&D), and 8 ng/ml bFGF (R&D) for one day. On days 2 and 3 cells were given the same medium, but without Wnt3A. On day 4, cultures were examined by flow cytometry for expression of CXCR4 as a marker of definitive endoderm. Cultures greater than 85% positive for CXCR4 were fed DMEM/F12 (D/F12; Life Technologies) containing 2% BSA, 50 ng/ml FGF7 (PeproTech, Inc., Rocky Hill, NJ, USA), 0.25 μM Cyclopamine-KAAD (CYC; Calbiochem, La Jolla, CA, USA) for days 4 through 6. Media for days 7 to 10 was D/F12 supplemented with 1% B-27 (Life Technologies), 50 ng/ml FGF7, 0.25 μM CYC, 0.2 μM retinoic acid (RA; Sigma-Aldrich Oakville, ON, Canada), and 100 ng/ml Noggin (PeproTech). Stage 4 media (day 11-13) was D/F12 + 1% B-27, 100 ng/ml Noggin, 1 μM ALK5 Inhibitor (ALK5inh; Alexis Biochemicals, San Diego, CA, USA), 1 μM DAPT (Calbiochem), and 100 ng/ml Netrin-4 (NET-4; R&D). Stage 5 media (day 14-21) was D/F12 + 1% B-27, and 1 μM ALK5inh. Between each differentiation stage the cells were washed twice with phosphate buffered saline without calcium or magnesium chloride (PBS-; Sigma) between media changes. On days 1, 4, 11, and 21 of the differentiation trial, cells seeded at each density were detached in triplicate by extended treatment with Accutase (5-15 minutes, 37°C, 5% CO_2_), washed once in PBS- and counted as a 1:10 diluted cell suspension using the Scepter^TM^ cell counter. 

 On day 19 of differentiation a one-hour sequential static glucose-stimulated hormone secretion assay was performed on differentiated cells. Briefly, the medium was aspirated and the cells were washed twice with PBS-, then incubated in 1.5 ml/well RPMI (Sigma, cat # 11879) containing 2 mM D-glucose (Sigma) at 37°C, 5% CO_2_ for two hours. Cells were incubated sequentially for one hour in RPMI + 2 mM glucose followed by RPMI + 25 mM glucose then RPMI + 30 mM potassium chloride. After each incubation period samples were collected, clarified of cell debris, and stored at -20°C for later assay. For similar assessment using radioimmunoassay (see below) twenty-four hour static media samples were taken at the end of each stage. 

### Flow Cytometry of Definitive Endoderm Induction and Cell Cycle Analysis

On the morning of day 4, differentiating cells were detached with Accutase for 8-10 minutes at 37°C, 5% CO_2_ and then washed twice in PBS- supplemented with 5% fetal bovine serum (FBS; Life Technologies). Cell pellets were resuspended in 300 μl of BD CytofixCytoperm (BD, cat # 554722) and incubated for 20 minutes at 4°C followed by two washes in BD Perm/Wash (BD, cat # 554723). Fixed and washed cells were stained with α-CXCR4-PE (R&D, 1:50), and α-SOX17-APC (R&D, 1:50) and/or relevant labelled isotype controls (R&D) diluted in BD Perm/Wash for 1 hour at room temperature. After two additional washes in BD Perm/Wash, cells were analyzed using an LSRII cytometer (Becton Dickinson, San Jose, CA, USA) for co-positive (CXCR4 and SOX17) cells relative to double isotype controls using FlowJo Software (Tree Star, Ashland, OR, USA).

**Figure 2:**
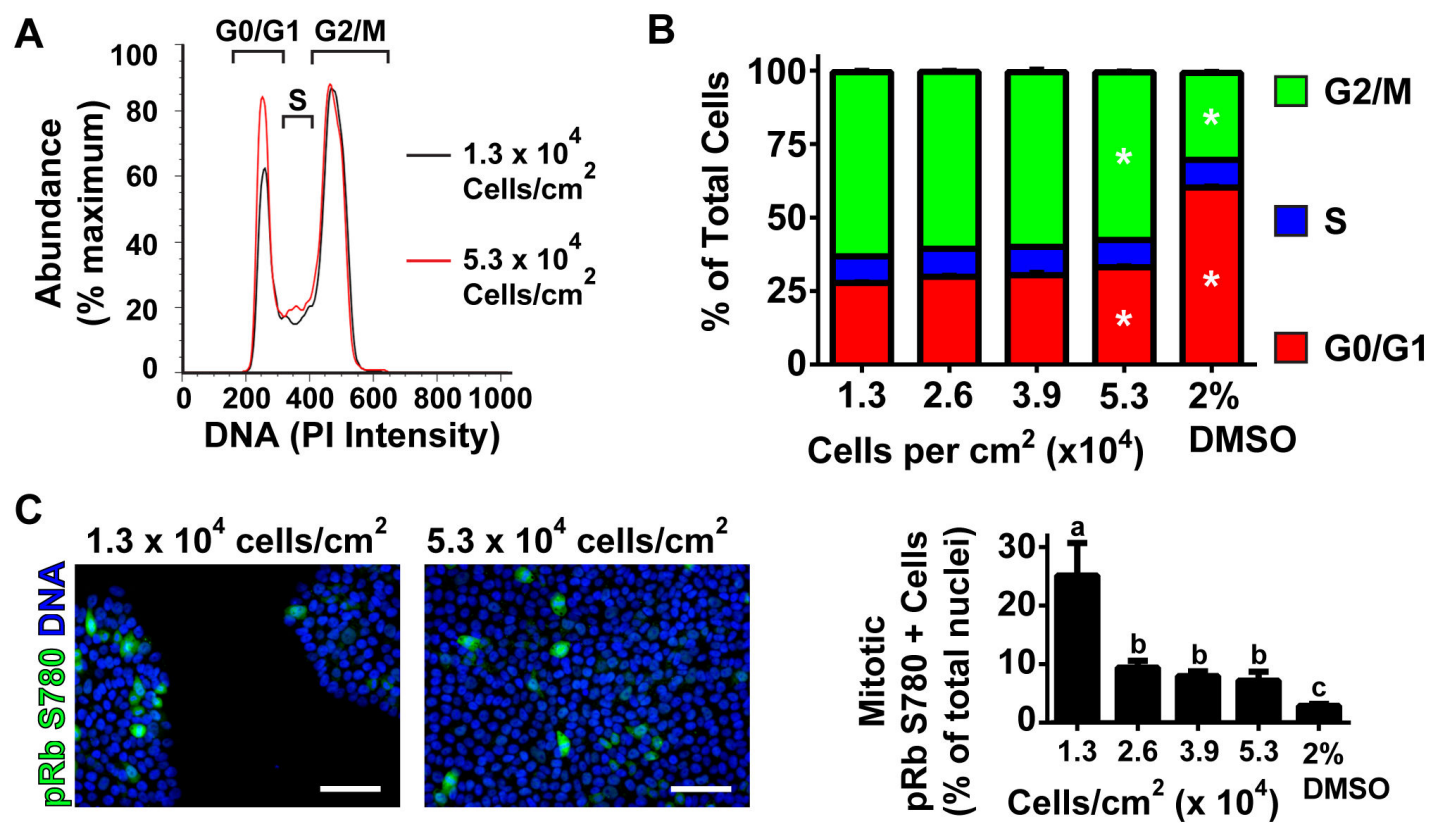
Higher Cell Seeding Density Decreases Cell Cycle Progression. (A) A representative histogram (left) of low density (1.3 x 10^4^ cells/cm^2^, black line) and high density (5.3 x 10^4^ cells/cm^2^, red line) seeded CA1S hESCs stained for DNA content by propidium iodide to indicate cell cycle state within the depicted gates 24-hours after seeding. (B) Single cells gated for uniform DNA width were assessed in triplicate and quantified as either G0/G1, S or G2/M phases using the gates in (A) as a percentage of the total single cell population. Four cell seeding densities of CA1S cells (1.3, 2.6, 3.9 and 5.3 x 10^4^ cells/cm^2^) along with 5.3 x 10^4^ cells/cm^2^ seeded cells treated overnight with 2% DMSO to induce cell cycle arrest (2% DMSO) were quantified. * represents significant difference from 1.3 x 10^4^ cells/cm^2^ by one-way ANOVA with Bonferroni post-hoc test within the same cell cycle population. (C) Representative images and quantification of immunocytochemistry of pRb S780 (green, nuclei are blue). pRb S780 positive mitotic cells were quantified as a percentage of the total cell populations in five randomly selected images. Different superscripts (a, b, c) are significantly different from each other by one-way ANOVA with Bonferroni post-hoc test. Scale bars are 100 μm.

 To determine cell cycle status prior to induction of definitive endoderm, undifferentiated CA1S and WA01 hESCs seeded at varying densities were dissociated with Accutase, washed twice in PBS- supplemented with 1% BSA, and fixed in 1% PFA in PBS- for 15 minutes on ice. After two washes in PBS- plus BSA, cells were resuspended in ice cold 80% ethanol added dropwise while vortexing before storage at -20°C. On the day of analysis, cells were washed twice with PBS- plus BSA and treated with 10 μg/ml RNAse A (SIGMA) in PBS- for 15 minutes at 37°C. Following two washes in PBS plus BSA, cells were incubated with 20 μg/ml propidium iodide (SIGMA) for 15 minutes at room temperature before analysis using a LSRII cytometer and FlowJo Software using standard gating strategies shown in Figure 2A. 

### Quantitative Reverse Transcriptase PCR (RT-qPCR)

Quantitative reverse transcriptase PCR (RT-qPCR) was performed according to the manufacturer's recommended protocols. Briefly, RNA was isolated using a RNeasy MiniKit (Qiagen, Hilden, Germany) including on-column DNAse digestion. cDNA was prepared from 250 ng RNA using iSCRIPT (BioRad, Hercules, CA, USA) and 2.5 ng of cDNA was used per qPCR reaction in SsoFast EvaGreen Supermix (BioRad) on a StepOnePlus instrument (Applied Biosystems, Foster City, CA, USA). Primers used for RT-qPCR can be found in Table S1. Unless otherwise stated, all RT-qPCR reactions were assayed in technical and biological triplicate with gene expression normalized first to its hypoxanthine-guanine phosphoribosyltransferase (HPRT) internal control, then to an external reference sample used to correct for plate-to-plate variation using the ΔΔCt method [27]. These external reference samples were pooled biological triplicates from different tissues including human liver, lung, pancreas (all from Life Technologies), and human islets (kindly provided by Dr. Ao and Dr. Warnock from the Irving K. Barber Human Islet Isolation Laboratory (Vancouver, BC, Canada) following appropriate ethical use approval).

### Immunocytochemistry

**Figure 3:**
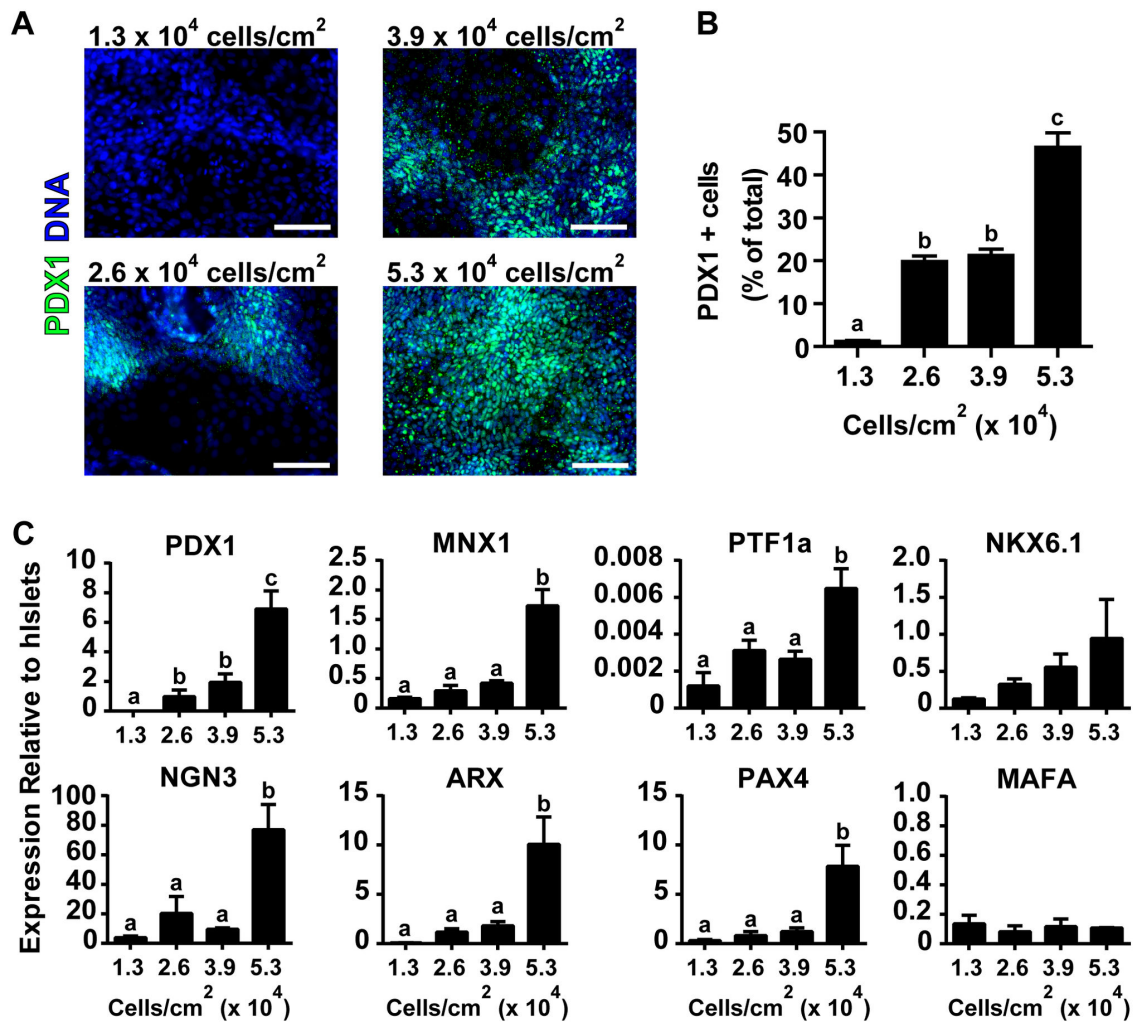
High Cell Seeding Density Increases Pancreatic Progenitor Differentiation. (A) hESCs seeded at different densities were differentiated for 14 days and immunostained for PDX1 (green) and DNA (blue). (B) Single-cell quantification of PDX1 positive nuclei as a percentage of total nuclei (C) RT-qPCR of 21 day differentiated cells. Expression is shown relative to isolated human islets. Different superscripts (a, b, c) are significantly different from each other within each graph by one-way ANOVA with Bonferroni post-hoc test. Scale bars are 100 μm.

**Figure 4:**
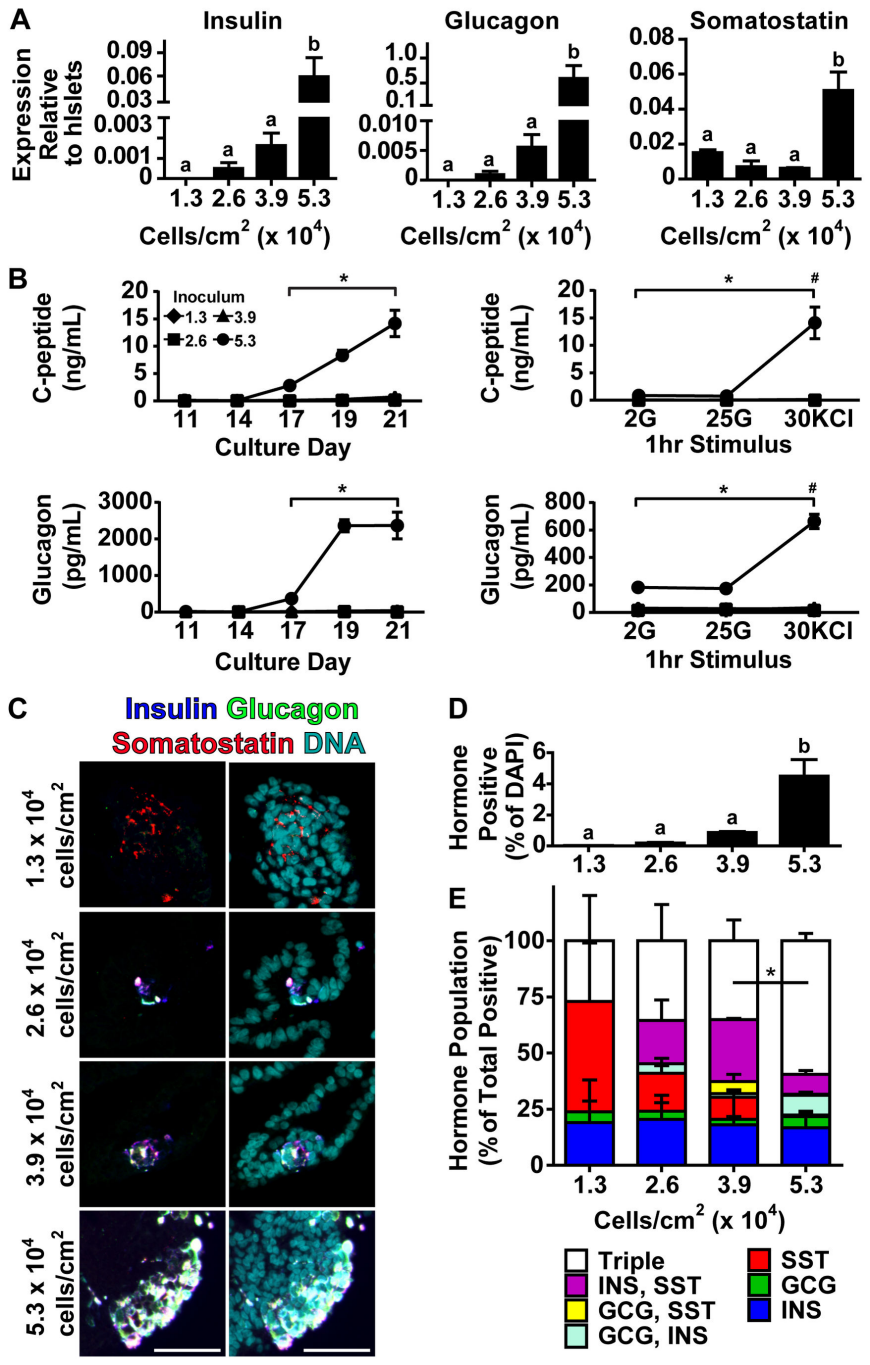
Higher Cell Seeding Density Enhances Pancreatic Endocrine Formation. (A) Insulin, glucagon, and somatostatin expression were assessed in 21 day differentiated hESCs using RT-qPCR (shown relative to human islets). (B) C-peptide and glucagon release were assayed in static 24 hour media samples take on the indicated culture, or during a sequential glucose and/or potassium chloride (KCl) stimulated hormone release assay performed on day 19 of culture. Following a 2 hour 2 mM glucose wash, cells were treated for 1 hour with 2 mM glucose (2G), 25 mM glucose (25G), then 30 mM KCl (30KCl). Diamonds, squares, triangles, and circles represent 1.3 x 10^4^ cells/cm^2^, 2.6 x 10^4^ cells/cm^2^, 3.9 x 10^4^ cells/cm^2^, and 5.3 x 10^4^ cells/cm^2^ initial seeding density respectively. * represents p<0.05 comparing 5.3 x 10^4^ cells/cm^2^ with other cell densities. # represents p<0.05 comparing KCl stimulation versus other stimuli within the 5.3 x 10^4^ cells/cm^2^ seeding density. (C) hESCs seeded at different densities and differentiated for 21 days were agarose-embedded and immunostained for insulin (blue), glucagon (green), somatostatin (red) and DNA (cyan). Right panel shows hormone staining and left panel shows the same hormone image with DNA. See Figure S4 for single channel images of a larger field of view. (D) Single-cell quantification of hormone population showing the number of cells positive for insulin, glucagon, or somatostatin as a percentage of the total number of nuclei. (E) Single-cell polyhormonal analysis of the hormone positive population in C as a percentage of total hormone positive population. Triple indicates cells scored positive for all three hormones. * represents p<0.05 comparing triple positive populations of 5.3 x 10^4^ cells/cm^2^ vs 3.9 x 10^4^ cells/cm^2^. In panels A and D, different superscripts (a, b) are significantly different from each other within each graph by one-way ANOVA with Bonferroni post-hoc test. Scale bars are 50 μm.

**Figure 5:**
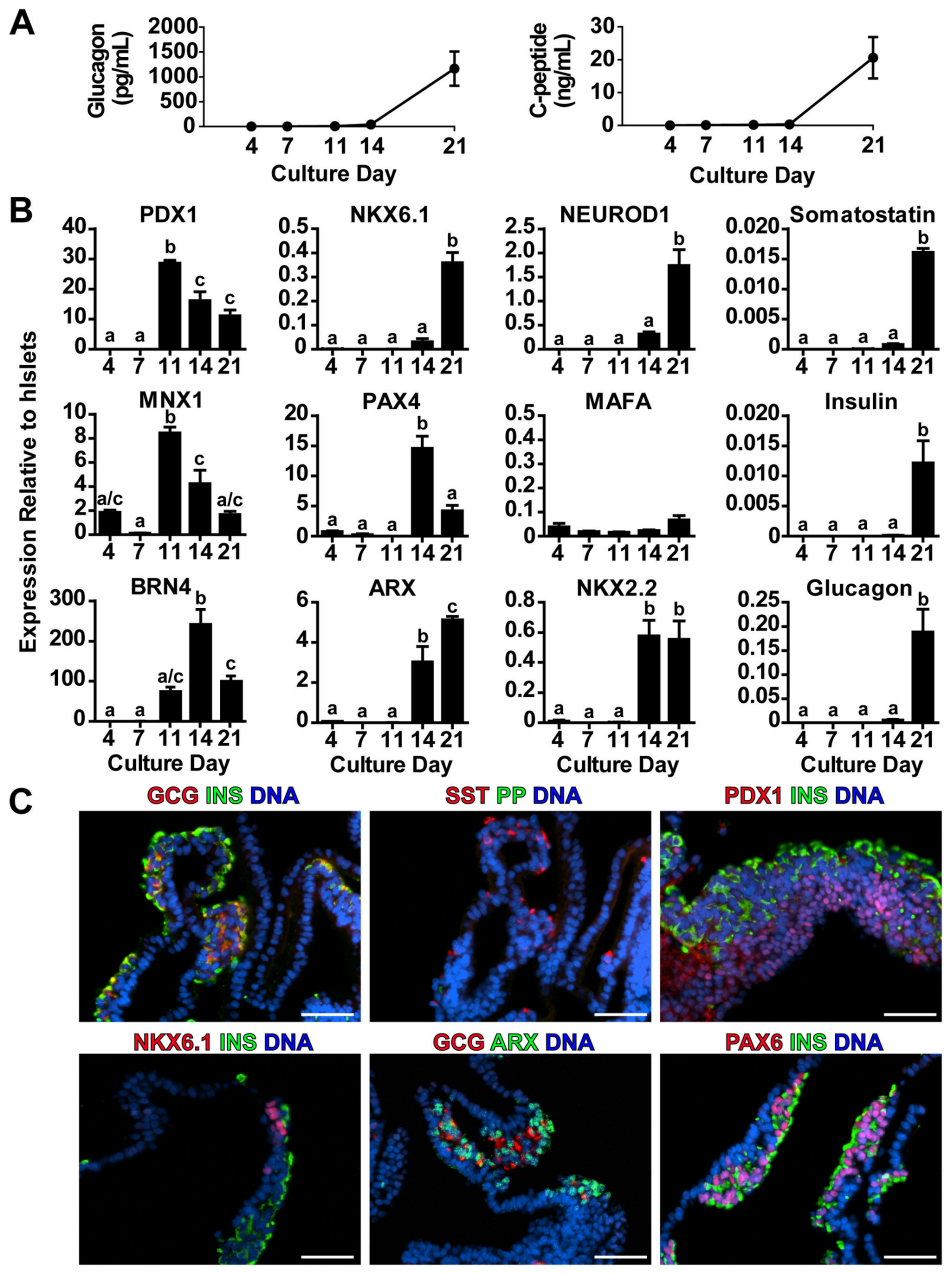
High Seeding Density Cultures Follow Expected Endocrine Developmental Timeline. (A) Media samples from multiple (N=12) high hESC cell seeding density differentiations contain reproducibly high levels of C-peptide and glucagon as measured by radioimmunoassay. (B) Over the differentiation time course expression of transcription factors and islet hormones was examined by RT-qPCR relative to adult human islet expression levels. (C) 21 day differentiated hESCs were immunostained as agarose-embedded, paraffinized sections for pancreatic hormones and key transcription factors involved in pancreatic endocrine induction and maturation. * represents p<0.05 comparing day 14 and 21 media content. Different superscripts (a, b, c) are significantly different from each other within each graph by one-way ANOVA with Bonferroni post-hoc test. Scale bars are 50 μm.

Differentiated cells were immunostained either directly in culture dishes (Figure 1, 2, 3 and Figure S2) or as sectioned cell pellets (Figure 4 and 5). For in-well staining, cells were fixed in 4% Paraformaldehyde (PFA) in PBS- overnight at 4°C, washed twice in PBS-, and permeabilized in 0.2% Triton X-100 (Sigma) in PBS- for 30 minutes at room temperature. After two more washes in PBS-, cells were incubated with primary antibodies (see Table S2) overnight at 4°C. After five sequential three minute washes in PBS-, Alexa 488-, 555-, or 647 conjugated secondary antibodies (Life Technologies; 1:1000 diluted in Dako Antibody diluent) were applied for 1 hour at room temperature followed by five, 3-minute washes in PBS- containing 2 ng/ml Hoechst 33342 (Life Technologies). For sectioned cell pellets, cultures were mechanically detached by scraping without enzymes. Detached cell sheets were transferred to 4% PFA in PBS- to fix overnight at 4°C. After two washes in PBS-, fixed cells were embedded in molten (50°C) 2% agarose (Life Technologies), chilled briefly on ice and fixed again in 4% PFA in PBS- for 1 hour at room temperature. Agarose embedded cell pellets were then stored in 70% ethanol prior to paraffinization and sectioning (Wax-it Histology Services, Vancouver, BC, Canada). Subsequent immunostaining of 5 μm sections on slides was performed as previously described [28] using primary antibodies described in Table S2 and appropriate secondary antibodies (Life Technologies). Imaging for both slides and cells in culture plates was performed using an ImageXpress Micro^TM^ automated microscope and associated software (Molecular Devices Corporation, Sunnyvale, CA, USA).

### Radioimmunoassay

Radioimmunoassays were performed on static media samples collected at the end of each differentiation stage as well as during the glucose-stimulated hormone release procedure. Both C-peptide (Millipore HCP-20K) and glucagon (Millipore GL-32K) were analyzed, following the manufacturer’s recommended protocols except using half volumes of all reagents. Analysis was performed in technical duplicate and biological triplicate. 

### Statistical Analysis

Data are reported as mean ± SEM with significance set at p ≤ 0.05 unless otherwise stated. Statistical comparisons were performed using one-way ANOVA and Bonferonni post-hoc tests.

## Results

### Definitive Endoderm Induction

To examine the effect of initial cell seeding density on subsequent differentiation to definitive endoderm and later pancreatic endocrine hormone-producing cells, we applied an established culture protocol (Figure 1A) known to yield polyhormonal pancreatic endocrine cells [18] from the CA1S hESC line. The CA1S line was chosen due to its previously described high seeding uniformity and capacity to form pancreatic endocrine cells [26]. Using this cell line we examined four seeding cell densities: 1.3 x 10^4^ cells/cm^2^, 2.6 x 10^4^ cells/cm^2^, 3.6 x 10^4^ cells/cm^2^, and 5.3 x 10^4^ cells/cm^2^, that corresponded to approximately 30%, 60%, 80%, and 100% confluence, respectively, by 16 hours after seeding (Figure 1B and Figure S1A). After 4 days of differentiation the cultures seeded at 3.6 x 10^4^ cells/cm^2^ and 5.3 x 10^4^ cells/cm^2^ had similar cell densities (Figure S1B). Induction of definitive endoderm was assessed by flow cytometry of CXCR4/SOX17 co-expression and RT-qPCR of *FOXA2* and Goosecoid. Cultures seeded at 2.6 x 10^4^ cells/cm^2^ or greater were found to contain significantly increased populations of CXCR4/SOX17 double positive cells as well as increased *FOXA2* and Goosecoid expression compared to those seeded at 1.3 x 10^4^ cells/cm^2^, suggesting enhanced definitive endoderm induction in cultures seeded at high density (Figure 1C). We examined the expression of OCT4, a marker of maintained pluripotent cell populations, by RT-qPCR and immunocytochemistry in order to assess whether cultures seeded at lower density remained arrested in the pluripotent state. Increased OCT4 levels were observed in cultures seeded at 1.3 x 10^4^ cells/cm^2^ at the end of stage 1 differentiation compared to cultures initially seeded at higher densities (Figure 1D). To examine whether this effect of cell density on definitive endoderm induction was specific to the CA1S hESC line, we applied a similar approach to the widely used WA01 (H1) hESC line. While WA01 cells required a higher initial cell seeding density and 48 hours of growth to achieve similar confluence as CA1S cells (Figure S2A) higher initial cell seeding density was associated with increased numbers of CXCR4/SOX17 double positive definitive endoderm cells. Conversely cultures of WA01 hESCs seeded at low density suffered from considerable cell losses during differentiation and the few cells which did survive were poorly specified to the endoderm germ layer (Figure S2B).

 To better understand how cell density potentially altered definitive endoderm induction we examined the role of cell cycle in both CA1S and WA01 hESCs at the start of our differentiation protocol. We found that CA1S and WA01 hESCs seeded at low cell density were biased toward the G2 and M phases of the cell cycle using DNA content assessment by flow cytometry (Figure 2A/B and Figure S2C/D). Similar to WA01 hESCs, CA1S cells seeded at low density cultures contained 27% cells in G0/G1 while high density cultures contained 33% cells in G0/G1 and cultures treated with 2% DMSO to induce cell cycle arrest contained 60% cells in G0/G1 (Figure 2B). Given the well established role of hyperphosphorylated retinoblastoma protein (pRb) in active cell cycle progression [29] we examined CA1S and WA01 hESCs seeded at different densities for pRb (serine 780) by immunocytochemistry. We found that undifferentiated hESCs stained brightly for pRb S780 including a fraction of cells that were in active mitosis as defined by DNA morphology and contained pRb S780 in the cytoplasm (Figure 2C and Figure S2E). Similar to WA01 hESCs, low density cultures of CA1S hESCs were found to contain significantly more of these cells (~25% of total cells) compared to high density cultures (~7% of total cells) and 2% DMSO treated hESCs (~3% of total cells). Together these data suggest that in high cell density cultures, similarly to DMSO treated cells, undergo a shift in cell cycle toward G0/G1 states. This shift is also associated with a decrease in cells that are undergoing active mitosis as marked by cytoplasmic pRb S780 positive cells. 

### Pancreatic Progenitor Restriction

After induction of definitive endoderm, cultures were continued to day 11; at this time point cultures seeded at 2.6 x 10^4^ cells/cm^2^ and above showed similar differentiated cell densities (Figure S1C). To examine the efficiency of pancreatic progenitor formation, PDX1 expression was examined by immunocytochemistry in day 14 cultures. Quantification of total nuclear PDX1 revealed a significant increase in PDX1 positive cells approaching 50% of the total cell population in cultures seeded at 5.3 x 10^4^ cells/cm^2^. The lowest seeding density culture (1.3 x 10^4^ cells/cm^2^), which demonstrated poor definitive endoderm induction, produced almost no PDX1 positive cells while the middle density cultures (2.6 x 10^4^ cells/cm^2^ and 3.9 x 10^4^ cells/cm^2^) contained approximately 20% PDX1 positive cells (Figure 3A and B). 

 Continued differentiation to day 21 revealed sustained expression of a number of pancreatic progenitor and endocrine fate specification transcription factors. *PDX1*, *MNX1*, and *PTF1a* expression was increased in cultures seeded at high density; *NKX6.1* expression also tended to be increased in high density cultures, although it did not reach statistical significance (Figure 3C). Similarly *NGN3*, *ARX*, and *PAX4* were increased in cultures seeded at 5.3 x 10^4^ cells/cm^2^ while *MAFA* expression levels did not differ amongst cultures seeded at different densities (Figure 3C). By day 21 culture density had plateaued at nearly 4 x 10^5^ cells/cm^2^ (Figure S1D) for cells seeded at 2.6 x 10^4^ cells/cm^2^ and above, possibly owing to the limited capacity of the standard adherent culture system used. 

 To examine cultures for unwanted non-endocrine differentiation, RT-qPCR was applied to cultures differentiated for 21 days to measure expression of albumin, *NKX2.1*, and amylase (markers of liver, lung, and exocrine pancreas, respectively). While albumin expression was significantly increased in cultures seeded at 2.6 x 10^4^ cells/cm^2^ compared to other seeding densities, no significant alternative cell fates predominated the cultures (Figure S3). 

### Pancreatic Endocrine Specification

Knowing that cultures seeded at high density contained increased numbers of PDX1 positive pancreatic progenitor populations, we next examined further differentiated cells for expression of pancreatic endocrine hormones using RT-qPCR. By day 21 of the differentiation protocol, cultures initially seeded at high density expressed significantly elevated levels of insulin, glucagon and somatostatin mRNA compared to cultures seeded at lower density (Figure 4A). We also assessed the content of glucagon and C-peptide, a marker of processed insulin, in 24-hour static media samples taken between days 11 and 21. Cultures seeded at 5.3 x 10^4^ cells/cm^2^ demonstrated significantly higher C-peptide and glucagon release from day 17 to 21 compared to all lower initial seeding densities (Figure 4B). Similarly, a sequential glucose or potassium chloride stimulation test carried out on day 19 of the differentiation protocol revealed that only the cultures seeded at 5.3 x 10^4^ cells/cm^2^ were able to release detectable C-peptide or glucagon in response to the stimuli tested. Importantly, the high glucose-stimulated C-peptide release and low glucose-stimulated glucagon release kinetics typical of a native human islet were not observed under any condition, and only potassium chloride was able to stimulate significant hormone release, suggesting immature cell populations (Figure 4B). 

 Since the 21 day differentiated cultures were producing pancreatic endocrine hormones, we next assessed whether the endocrine cell populations were producing single hormones (suggesting maturation), or were polyhormonal, suggesting immaturity in line with what is thought to occur during human fetal development [30-32]. Agarose-embedded, paraffinized sections of 21 day cultures were immunostained for insulin, glucagon, and somatostatin (Figure 4C and Figure S4). Nucleocentric automated cell scoring revealed that cultures seeded at high density had increased numbers of cells that were positive for any combination of these hormones approaching 6% of the total DAPI positive cell population (Figure 4D). While all four seeding densities had the capacity to induce formation of unihormonal, bihormonal, or trihormonal subpopulations of cells, cultures from the highest initial seeding density produced mostly polyhormonal cells (insulin, glucagon, and somatostatin positive), which tended to cluster together (Figure 4C and Figure S4).

 Given that high initial cell seeding density seemed to promote increased numbers of immature polyhormonal cells, we were interested in whether expression of transcription factors in these cultures followed the expected temporal patterns of human fetal gene expression during hESC differentiation. Numerous subsequent differentiation trials using high initial seeding densities recapitulated the progressive release of glucagon and C-peptide into culture media during the differentiation period (Figure 5A). Expression of pancreatic progenitor markers *PDX1* and *MNX1* were upregulated at day 11, while expression of endocrine fate specification factors *BRN4*, *NKX2.2*, *ARX* and *PAX4* was observed around day 14. Expression levels of maturation factors *NKX6.1* and *NEUROD1* were enhanced at day 21, coincident with insulin, glucagon and somatostatin expression (Figure 5B). However, *MAFA* expression was not observed, suggesting a lack of maturation in all of these cell populations. We also examined final cell populations for expression of some of these transcription factors using immunocytochemistry. We again observed polyhormonal cells predominating the cultures with more rare unihormonal cells. Expression of PAX6 and ARX was observed in developing endocrine (insulin or glucagon positive) clusters, while abundant PDX1 staining and rare NKX6.1 staining was observed in a separate cell compartment not positive for insulin on glucagon (Figure 5C). 

## Discussion

In this study we examined the effect of modifying initial cell seeding density at the start of pancreatic endocrine differentiation of hESCs. We predominantly used the CA1S hESC line, which allows uniform and highly reproducible cell seeding at a number of densities without loss of pluripotency [26]. Upon differentiation, we observed an early failure to commit to definitive endoderm in cultures seeded at low density. These cultures contained a small fraction (~30%) of cells that were copositive for CXCR4 and SOX17 compared to nearly 75% copositive fractions in cultures seeded at higher density. Further examination of the low density cultures revealed remaining OCT4 positive cells, which had presumably failed to differentiate under the conditions that were suitable for definitive endoderm induction in cultures seeded at higher density (Figure 1C and D). This suggests that despite the availability of differentiation signals, cultures seeded at low density were apparently unable to fully convert from the pluripotent gene expression programme to one of definitive endoderm expression. One possible reason for this failure is the increased number of cells in the G2/M phases of the cell cycle associated with hyperphosphorylation of retinoblastoma protein (Figure 2 and Figure S2). As previous studies have noted, hESCs are amenable to differentiation during the G1 phase of cell cycle and prefer to remain undifferentiated during the G2 and M phases [33,34]. In the cultures seeded at higher density (2.6 x 10^4^ cells/cm^2^ and above) we observed a bias away from the G2/M phases of the cell cycle with decreased phosphorylation of retinoblastoma protein and a threshold effect of efficient definitive endoderm induction including expression of SOX17, CXCR4, Gooscoid, *FOXA2* and low OCT4. Taken together these data suggest a link between the cell cycle status of hESCs at the start of differentiation and the efficiency of definitive endoderm induction four days later. 

 As differentiation continued between days 4 and 21, the CA1S cultures, initially seeded at variable density, grew to the apparent capacity of the 12-well culture system (Figure S1). During this time period the process of sequential maturation from definitive endoderm (Figure 1) through pancreatic progenitors (Figure 3) to polyhormonal pancreatic endocrine cells (Figure 4) followed a temporal cascade of transcription factor expression (Figure 5). Based on the order of transcription factor expression, the cultures seemed to follow a trajectory of pancreatic progenitors expressing *PDX1* and *MNX1*, followed by endocrine specification with expression of *BRN4*, *ARX*, *PAX4*, *NKX6.1*, and *NEUROD1*, and eventually expression of insulin, glucagon, and somatostatin (Figure 5B). This expression pattern follows many of the transcription factor mediated developmental pathways believed to drive formation of pancreatic endocrine cells in humans and mice [3,11,35,36]. Notably, *MAFA* expression and glucose stimulated insulin secretion was not observed under any of the seeding densities we tested (Figure 4B) or at any differentiation timepoint (Figure 5B). Given that MAFA expression is believed to be critical for proper insulin secretion kinetics in mice [37,38], and has been observed in adult human beta cells but not in immature human fetal endocrine cells [30], it is perhaps not surprising that the endocrine cells produced in this study did not exhibit mature capacity for glucose-induced insulin release. The immaturity of the endocrine cells produced in this study is in line with previously published *in vitro* differentiation results which report the formation of polyhormonal cells that release C-peptide in response to potassium chloride depolarization but are not capable of the mature glucose regulated insulin secretion observed with native islets [39]. While this does not preclude the presence of mature beta-cells within the cultures, such cells likely make up a small fraction of the total cell population at 21 days of differentiation, although they may increase in number during extended culture [18]. 

 During previous work in which the role of retinoic acid on pancreatic and liver progenitor formation was examined using an enzymatic dissociation and replating strategy, Cai et al. (2010) observed that retinoic acid dependent PDX1 expression was increased when cells were seeded at higher densities [21]. While not the primary finding, the study implicated cell density as a contributing factor enabling retinoic acid to promote PDX1 expression. The enzymatic and mechanical dissociation protocol used to control cell density is not ideal for a simplified scalable differentiation protocol and warranted further examination. Our data show that even initial seeding density can affect PDX1 expression in differentiating hESCs even without further mechanical dissociation during the differentiation protocol. This notion is also supported by the recent work of Chetty et al. [24]. In their work higher initial cell seeding density was found to increase the number of SOX17 positive definitive endoderm cells as well as later PDX1 positive cell populations. Furthermore, high density seeded cultures had increased numbers of cells in the G1 phase of the cell cycle, which was associated with hypophosphorylation of the retinoblastoma protein implicating cell cycle progression as a key aspect of hESC differentiation capacity. The current study indicates that initial seeding density also impacts the formation of definitive endoderm, PDX1 positive pancreatic progenitors, and eventual hormone positive cells arising from hESCs. While the mechanism of action for this effect seems to be related to the cell cycle status of the initial seeded population and specifically the phosphorylation status of retinoblastoma protein (this study and others [24,33,34]), the link between extracellular cell-cell interactions and the associated pause in the G1 phase of the cell cycle is not completely clear. hESCs are known to be highly proliferative, with a particularly short G1 cell cycle, minimal check point control and decreased sensitivity to extracellular cues [40-43]. Cell density likely plays a key role regulating cell-cell interactions which prime hESCs to be receptive to instructive differentiation signals. This priming effect seems to be linked to decreased proliferation allowing key pancreatic developmental checkpoints to be efficiently achieved.

 Ultimately, the goal of this study was to determine the effect of initial cell seeding density on pancreatic endocrine differentiation of hESCs. This variable, inherent to nearly all cell culture processes, was found to have marked effects at every differentiation stage examined including germ layer induction, pancreatic progenitor restriction, and endocrine specification with the notable exception of functional *in vitro* maturation. In efforts to produce glucose responsive insulin-positive cells from hESCs, it seems likely that as signalling pathways that control hESC development are identified, their efficiency of action will be dependent on the culture conditions to which the cells are exposed. While cell density was examined in this study, other factors including temperature, oxygen tension, pH, osmolarity, and metabolite compositions are candidates that should be examined to modify the formation of pancreatic endocrine cells from hESCs. Optimization of these simple factors could increase the yield of pancreatic progenitor and endocrine cells from differentiation protocols, while improving our understanding of hESC development and advancing the possibility of a clinical scale therapeutic product for diabetes. 

## Supporting Information

Figure S1 **Cell Density Tracking Over Differentiation.** Differentiating CA1S hESCs were counted at a series of time points during culture following complete enzymatic dissociation and automated cell counting. (A) hESC cell counts 24 hours after seeding at the indicated cell inoculums at the time just prior to starting the differentiation protocol. (B) 4 day differentiated cell counts at the time of analysis for markers of definitive endoderm. (C) 11 day differentiated cell counts at the end of stage 3. (D) 21 day cell counts at the end of stage 5 at the termination of the differentiation protocol. Different superscripts (a, b, c, d) are significantly different from each other within each graph by one-way ANOVA with Bonferroni post-hoc test.
(TIF)

Figure S2 **High Cell Seeding Density Improves Definitive Endoderm Differentiation and is Associated with Decreased Cell Cycle Progression in WA01 hESCs .** (A) WA01 hESCs were seeded onto matrigel-coated plates at the indicated densities, allowed to expand for 48 hours (day 1) and differentiated to definitive endoderm (day 4) following the protocol in Figure 1A. (B) On day 4 of differentiation, markers of definitive endoderm induction were assessed by flow cytometry (CXCR4 and SOX17 expression as a percentage of the total single cell fraction). (C) A representative histogram (left) of low density (2.6 x 10^4^ cells/cm^2^, black line) and high density (10.6 x 10^4^ cells/cm^2^, red line) seeded WA01 hESCs stained for DNA content by propidium iodide to indicate cell cycle state within the depicted gates 48-hours after seeding. (D) Single cells gated for uniform DNA width were assessed in triplicate and quantified as either G0/G1, S or G2/M phases using the gates in (C) as a percentage of the total single cell population. Four cell seeding densities of WA01 cells (2.6, 5.2, 7.8 and 10.6 x 10^4^ cells/cm^2^) were examined for cell cycle status. (E) Representative images and quantification of immunocytochemistry of pRb S780 (green, nuclei are blue). pRb S780 positive mitotic cells were quantified as a percentage of the total cell populations in five randomly selected images. * represents significant difference from 2.6 x 10^4^ cells/cm^2^ by one-way ANOVA with Bonferroni post-hoc test within the same population. Different superscripts (a, b, c) are significantly different from each other by one-way ANOVA with Bonferroni post-hoc test. Scale bars are 100 μm.
(TIF)

Figure S3 **Cell Seeding Density Affects Off Target Differentiation.** RT-qPCR of 21 day differentiated cells. Expression relative to human liver (Albumin), human lung (NKX2.1), or human pancreas (Amylase). Different superscripts (a, b) are significantly different from each other within each graph by one-way ANOVA with Bonferroni post-hoc test.
(TIF)

Figure S4 **Polyhormonal Pancreatic Endocrine Cells.** hESCs seeded at different densities and differentiated for 21 days were agarose-embedded and immunostained for insulin (blue), glucagon (green), somatostatin (red) and DNA (cyan). Individual colour channels for red, green, and blue as well as three colour merge and three colour merger with DNA is laid out from left to right. White colour depicts colocalization in cells immunoreactive for all three hormones in the merged series. * denotes approximate region depicted in Figure 4C. Scale bar is 100 μm.
(TIF)

Table S1 **RT-qPCR Primers.** Primers, product sizes and references where applicable for indicated genes examined in this study.
(PDF)

Table S2 **Antibody Sources and Conditions for Immunocytochemistry.** Antibody sources and information associated with staining conditions are provided for proteins examined in this study.
(PDF)
