# Supplementary figures and images for: Initial Cell Seeding Density Influences Pancreatic Endocrine Development During in vitro Differentiation of Human Embryonic Stem Cells
Source: PLoS One. 2013 Dec 4;8(12):e82076. doi: 10.1371/journal.pone.0082076 (PMC3852888; doi:10.1371/journal.pone.0082076)

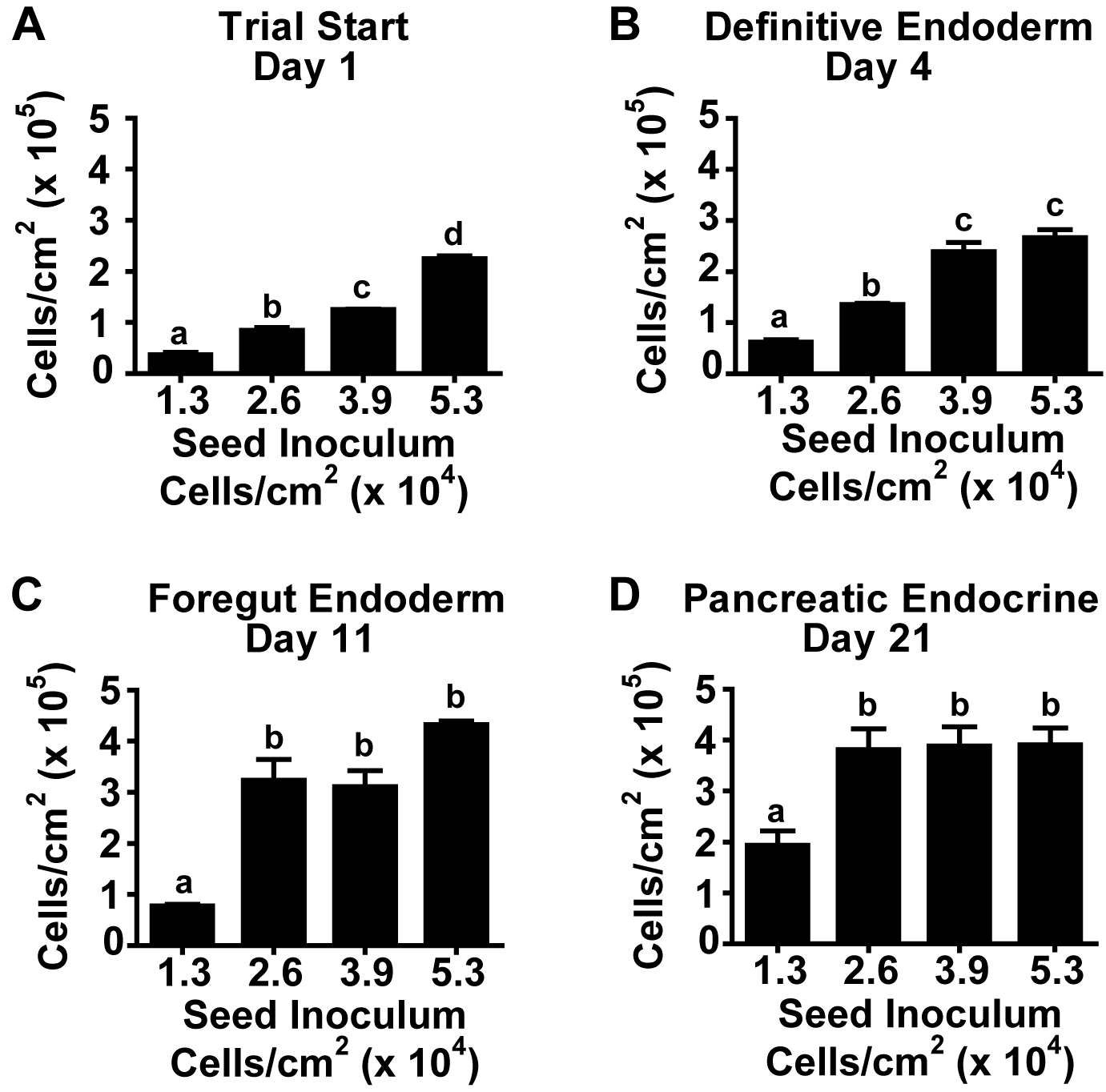

Supplement: Figure S1 — Cell Density Tracking Over Differentiation. Differentiating CA1S hESCs were counted at a series of time points during culture following complete enzymatic dissociation and automated cell counting. (A) hESC cell counts 24 hours after seeding at the indicated cell inoculums at the time just prior to starting the differentiation protocol. (B) 4 day differentiated cell counts at the time of analysis for markers of definitive endoderm. (C) 11 day differentiated cell counts at the end of stage 3. (D) 21 day cell counts at the end of stage 5 at the termination of the differentiation protocol. Different superscripts (a, b, c, d) are significantly different from each other within each graph by one-way ANOVA with Bonferroni post-hoc test. (TIF) [file pone.0082076.s001.tif]

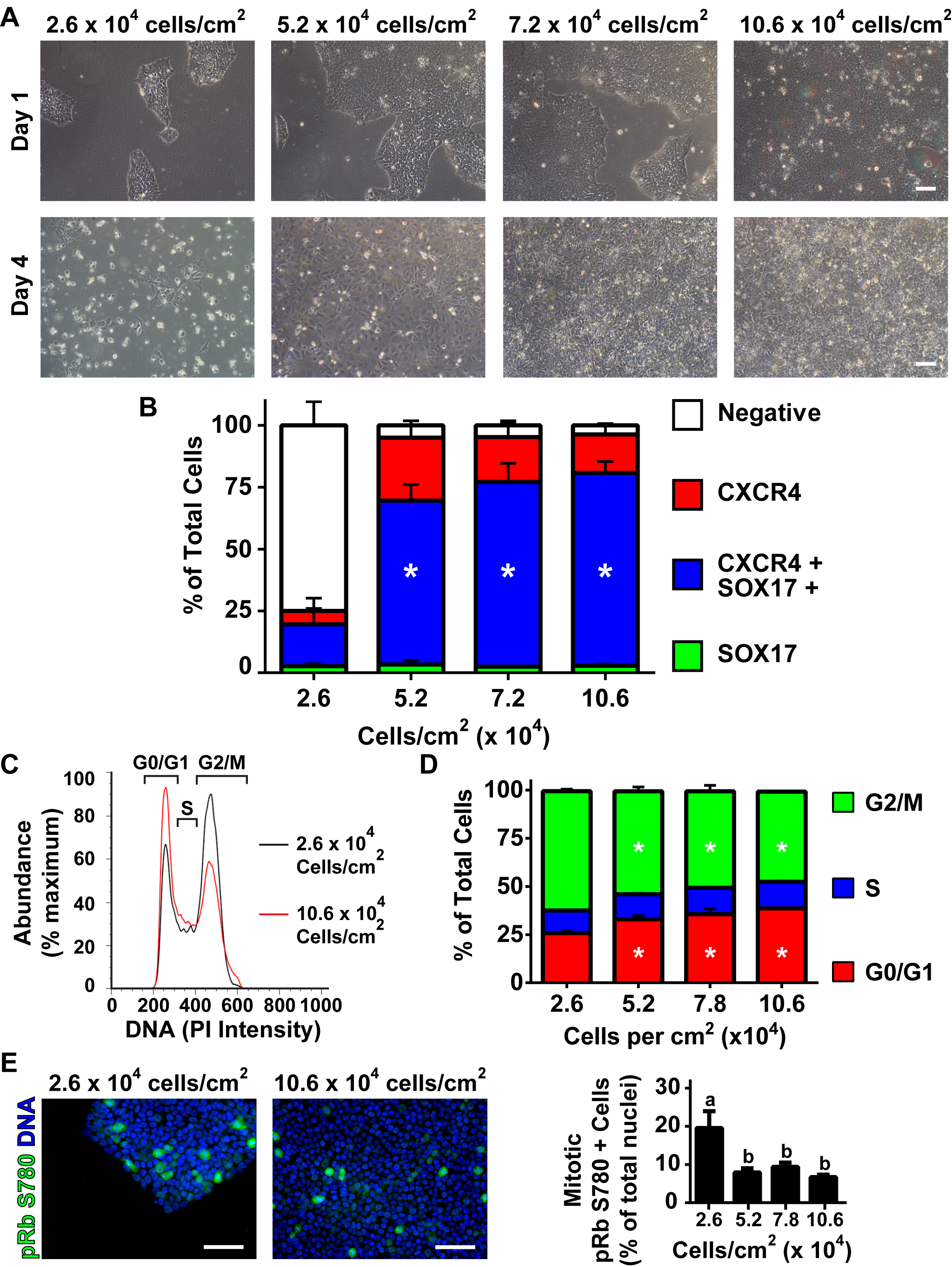

Supplement: Figure S2 — High Cell Seeding Density Improves Definitive Endoderm Differentiation and is Associated with Decreased Cell Cycle Progression in WA01 hESCs . (A) WA01 hESCs were seeded onto matrigel-coated plates at the indicated densities, allowed to expand for 48 hours (day 1) and differentiated to definitive endoderm (day 4) following the protocol in Figure 1A. (B) On day 4 of differentiation, markers of definitive endoderm induction were assessed by flow cytometry (CXCR4 and SOX17 expression as a percentage of the total single cell fraction). (C) A representative histogram (left) of low density (2.6 x 10^4^ cells/cm2, black line) and high density (10.6 x 104 cells/cm2, red line) seeded WA01 hESCs stained for DNA content by propidium iodide to indicate cell cycle state within the depicted gates 48-hours after seeding. (D) Single cells gated for uniform DNA width were assessed in triplicate and quantified as either G0/G1, S or G2/M phases using the gates in (C) as a percentage of the total single cell population. Four cell seeding densities of WA01 cells (2.6, 5.2, 7.8 and 10.6 x 104 cells/cm2) were examined for cell cycle status. (E) Representative images and quantification of immunocytochemistry of pRb S780 (green, nuclei are blue). pRb S780 positive mitotic cells were quantified as a percentage of the total cell populations in five randomly selected images. * represents significant difference from 2.6 x 10^4^ cells/cm2 by one-way ANOVA with Bonferroni post-hoc test within the same population. Different superscripts (a, b, c) are significantly different from each other by one-way ANOVA with Bonferroni post-hoc test. Scale bars are 100 μm. (TIF) [file pone.0082076.s002.tif]

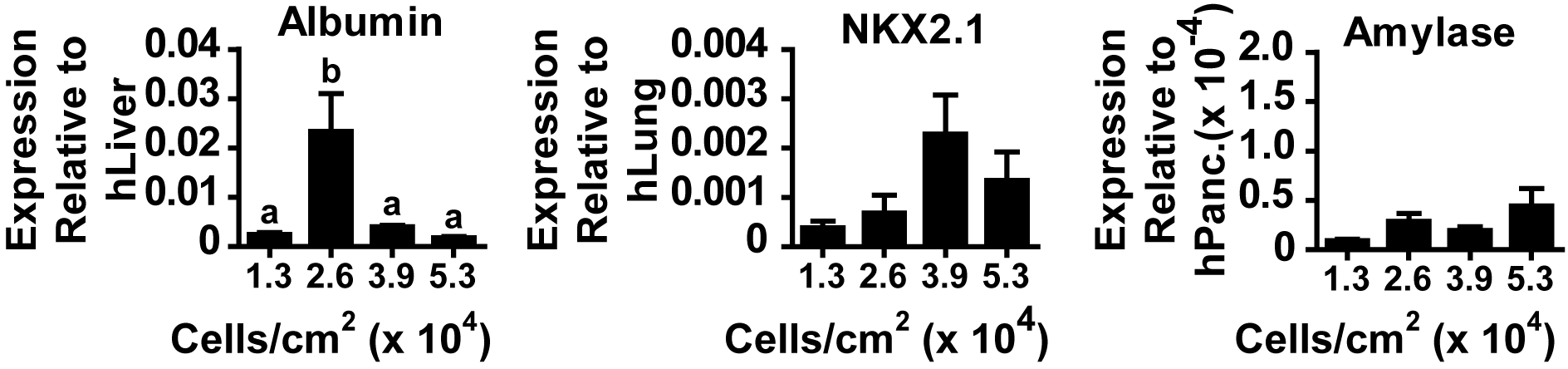

Supplement: Figure S3 — Cell Seeding Density Affects Off Target Differentiation. RT-qPCR of 21 day differentiated cells. Expression relative to human liver (Albumin), human lung (NKX2.1), or human pancreas (Amylase). Different superscripts (a, b) are significantly different from each other within each graph by one-way ANOVA with Bonferroni post-hoc test. (TIF) [file pone.0082076.s003.tif]

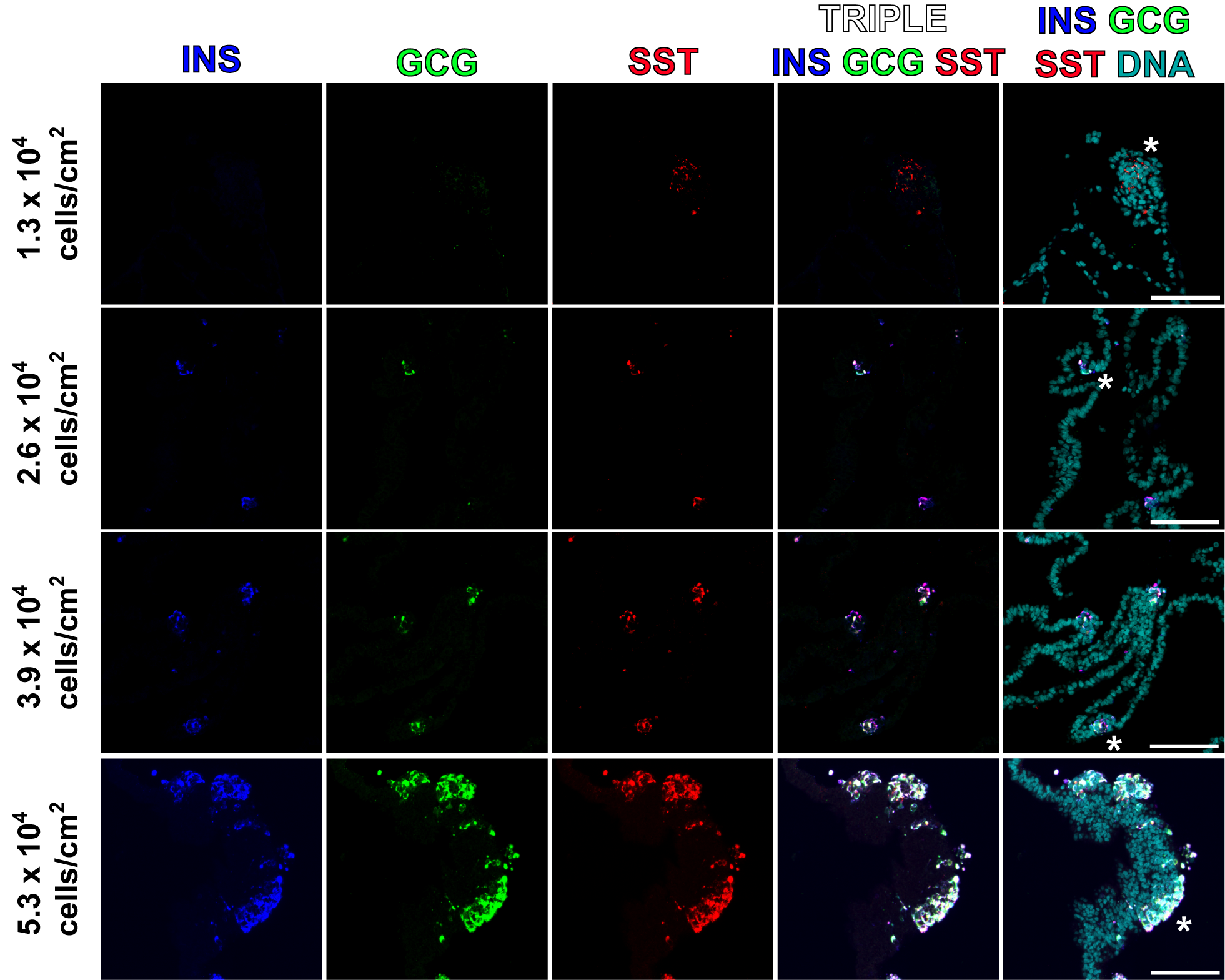

Supplement: Figure S4 — Polyhormonal Pancreatic Endocrine Cells. hESCs seeded at different densities and differentiated for 21 days were agarose-embedded and immunostained for insulin (blue), glucagon (green), somatostatin (red) and DNA (cyan). Individual colour channels for red, green, and blue as well as three colour merge and three colour merger with DNA is laid out from left to right. White colour depicts colocalization in cells immunoreactive for all three hormones in the merged series. * denotes approximate region depicted in Figure 4C. Scale bar is 100 μm. (TIF) [file pone.0082076.s004.tif]
