# Supplementary material for: Initial Cell Seeding Density Influences Pancreatic Endocrine Development During in vitro Differentiation of Human Embryonic Stem Cells
Source: PLoS One. 2013 Dec 4;8(12):e82076. doi: 10.1371/journal.pone.0082076 (PMC3852888; doi:10.1371/journal.pone.0082076)
Supplement: Table S1 — RT-qPCR Primers. Primers, product sizes and references where applicable for indicated genes examined in this study. (PDF) [file pone.0082076.s005.pdf]

Table S1 - RT-qPCR primers

Gene Name	Gene Accession	Product Size (bp)	Primer Sequence Forward / Reverse 5'→3'	Ref.
OCT4	NM_002701.4	78	TGGGCTCGAGAAGGATGTG<br>GCATAGTCGCTGCTTGATCG	[1]
FOXA2	NM_021784.4	89	GGGAGCGGTGAAGATGGA<br>TCATGTTGCTCACGGAGGAGTA	[1]
Goosecoid	NM_173849.2	70	GAGGAGAAAGTGGAGGTCTGGTT<br>CTCTGATGAGGACCGCTTCTG	[1]
PDX1	NM_000209	178	CGTCCAGCTGCCTTTCCCAT<br>CCGTGAGATGTACTTGTTGAATAGGA	[2]
MNX1	NM_005515.3	115	TCGCTCATGCTCACCGAGA<br>CCTTCTGTTTCTCCGCTTCCT	
PTF1a	NM_178161	180	GCAGCCAGGCCCAGAAGGTC<br>TTCTGGGGTCCTCTGGGGTCCA	
NKX6.1	NM_006168	186	GCCCGCCCTGGAGGGACGCA<br>ACGAATAGGCCAAACGAGCCC	[2]
NGN3	NM_020999	286	AGACGACGCGAAGCTCACC<br>AAGCCAGACTGCCTGGGCT	
ARX	NM_139058.2	141	CTGCTGAAACGCAAACAGAGGC<br>CTCGGTCAAGTCCAGCCTCATG	
PAX4	NM_006193	169	AGCAGAGGCACTGGAGAAAGAGTT<br>CAGCTGCATTTCCCACTTGAGCTT	[2]
MAFA	NM_201589	195	CTTCAGCAAGGAGGAGGTCA<br>TTGTACAGGTCCCGCTCTTT	
Insulin	NM_000207.2	245	AGCCTTTGTGAACCAACACC<br>GCTGGTAGAGGGAGCAGATG	[2]
Glucagon	NM_002054.4	275	CATTCACAGGGCACATTCAC<br>CGGCCAAGTTCTTCAACAAT	[2]
Somatostatin	NM_001048.3	126	AGCTGCTGTCTGAACCCAAC<br>CCATAGCCGGGTTTGAGTTA	[2]
NeuroD1	NM_002500.2	146	GCCCCAGGGTTATGAGACTAT<br>GAGAACTGAGACACTCGTCTGT	
BRN4	NM_000307.3	150	CTGCAACTGGGTGCGATCAT<br>AGGCTGCGAGTACACGTTGA	
NKX2.2	NM_002509	221	CTTCTACGACAGCAGCGACAACCCG<br>CCTTGGAGAAAAGCACTCGCCGCTTT	[2]
Albumin	NM_000477.5	216	CCTTGGTGTTGATTGCCTT<br>TTGCACAGCAGTCAGCCAT	[3]
NKX2.1	NM_001079668.2	164	GTACCAGGACACCATGAGGAAC<br>CCATGTTCTTGCTCACGTCCC	[4]
Amylase	NM_000699.2	141	AATGTCAAGCTACCGTTGGCC<br>TTCACAGACCCAGTCATTGCC	[3]
HPRT	NM_000194.2	148	TGTTGTAGGATATGCCCTTGACTAT<br>GCGATGTCAATAGGACTCCAGA	

References

1. D'Amour KA, Agulnick AD, Eliazer S, Kelly OG, Kroon E, et al. (2005) Efficient differentiation of human embryonic stem cells to definitive endoderm. Nat Biotechnol 23: 1534-1541.
2. Gage BK, Riedel MJ, Karanu F, Rezania A, Fujita Y, et al. (2010) Cellular reprogramming of human amniotic fluid cells to express insulin. Differentiation 80: 130-139.
3. Mfopou JK, Chen B, Mateizel I, Sermon K, Bouwens L (2010) Noggin, retinoids, and fibroblast growth factor regulate hepatic or pancreatic fate of human embryonic stem cells. Gastroenterology 138: 2233-2245, 2245 e2231-2214.
4. Mou H, Zhao R, Sherwood R, Ahfeldt T, Lapey A, et al. (2012) Generation of multipotent lung and airway progenitors from mouse ESCs and patient-specific cystic fibrosis iPSCs. Cell Stem Cell 10: 385-397.
