# Supplementary material for: Initial Cell Seeding Density Influences Pancreatic Endocrine Development During in vitro Differentiation of Human Embryonic Stem Cells
Source: PLoS One. 2013 Dec 4;8(12):e82076. doi: 10.1371/journal.pone.0082076 (PMC3852888; doi:10.1371/journal.pone.0082076)
Supplement: Table S2 — Antibody Sources and Conditions for Immunocytochemistry. Antibody sources and information associated with staining conditions are provided for proteins examined in this study. (PDF) [file pone.0082076.s006.pdf]

Table S2 - Antibody Sources and Conditions for Immunocytochemistry

Gene Name	Host Species	Supplier / Catalogue number	Staining Method	Dilution	Antigen Retrieval
CXCR4	Mouse IgG2A - PE	R&D Systems FAB170P	Fixed cell Flow	1:50	None
SOX17	Goat IgG APC	R&D Systems IC1924A	Fixed cell Flow	1:50	None
OCT4	Goat	R&D Systems AF1759	4% PFA fixed monolayer	1:500	None
PDX1	Guinea Pig	Abcam Ab47308	4% PFA fixed monolayer	1:250	None
pRb S780	Rabbit	Cell Signalling 9307	4% PFA fixed monolayer	1:350	None
PDX1	Guinea Pig	Abcam Ab47308	PFA fixed, paraffin section of agarose embedded cell pellet	1:1000	HIER
PDX1	Rabbit	Dr. J. Habener Gift	PFA fixed, paraffin section of agarose embedded cell pellet	1:1000	HIER
Insulin	Guinea Pig	Sigma I8510	PFA fixed, paraffin section of agarose embedded cell pellet	1:1000	HIER
Glucagon	Rabbit	Thermo Scientific PA1-37768	PFA fixed, paraffin section of agarose embedded cell pellet	1:200	HIER
Glucagon	Mouse	Sigma G2654	PFA fixed, paraffin section of agarose embedded cell pellet	1:1000	HIER
Somatostatin	Mouse	BCBC AB1985	PFA fixed, paraffin section of agarose embedded cell pellet	1:1000	HIER
Pancreatic Polypeptide	Goat	R&D Systems AF6297	PFA fixed, paraffin section of agarose embedded cell pellet	1:200	HIER
NKX6.1	Rabbit	Dr. A. Rezania Gift	PFA fixed, paraffin section of agarose embedded cell pellet	1:1000	HIER
ARX	Rabbit	Dr. P. Collombat Gift	PFA fixed, paraffin section of agarose embedded cell pellet	1:500	HIER
PAX6	Rabbit	Covance PRB-278P	PFA fixed, paraffin section of agarose embedded cell pellet	1:250	HIER

HIER (heat induced epitope retrieval): 15 minutes at 95°C in 10mM Citrate buffer with 0.05% Tween-20 pH 6.0.
BCBC (Beta Cell Biology Consortium)

Affiliations of Antibody Gifts:
Dr. Habener: Laboratory of Molecular Endocrinology; Massachusetts General Hospital; Boston, MA USA
Dr. Rezania: BetaLogics Venture, Janssen R&D LLC, Raritan, New Jersey, USA.
Dr Collombat: Université de Nice-Sophia Antipolis, Inserm U1091, IBV, Diabetes Genetics Team, FR-06108 Nice, France.
